# Occlusion intestinale aigue révélant un lymphome T digestif associé à la maladie cœliaque, à propos d'un cas

**DOI:** 10.11604/pamj.2016.23.48.8909

**Published:** 2016-02-19

**Authors:** Abdoul Aziz Garba, Harissou Adamou, Ibrahim Amadou Magagi, Souleymane Brah, Oumarou Habou

**Affiliations:** 1 Service de Médecine Interne et Générale, Hôpital National de Zinder, Niger; 2 Service de Chirurgie Générale et Digestive B, Hôpital National de Zinder, Niger; 3 Service de Médecine Interne et Générale, Hôpital National de Niamey, Niger

**Keywords:** Maladie cœliaque, lymphome T, EATL, occlusion intestinale, chirurgie, Celiac disease, T-cell lymphoma, EATL, bowel obstruction, surgery

## Abstract

Le lymphome T intestinal associé à une entéropathie ou Enteropathy associated T-cell lymphoma (EATL), est une complication rare de la maladie cœliaque (MC). Nous rapportons l'observation d'un lymphome T associée à une MC révélé par une occlusion intestinale aigue. Une patiente maghrébine de 38 ans, aux antécédents de stérilité et de douleurs abdominales chroniques, était admise en urgence pour occlusion intestinale aigue. L'intervention chirurgicale retrouvait une tumeur au dépend du grêle avec des adénopathies mésentériques. L'histologie et l'immunohistochimie de la pièce opératoire objectivait un lymphome T digestif CD3+ et le bilan immunologique de la maladie cœliaque était positif. Le diagnostic d'EATL était ainsi retenu. La patiente était mise sous chimiothérapie (CHOEP) et régime sans gluten avec une réponse complète au traitement. L'EATL est une complication rare de la MC qui peut être révélée par une occlusion intestinale. Son pronostic peut être amélioré par une prise en charge précoce associant chirurgie et chimiothérapie. Sa prévention passe par un diagnostic précoce de la MC et un régime sans gluten.

## Abstract (en)

Enteropathy associated T-cell lymphoma (EATL) is a rare complication of celiac disease (CD). We report a case of EATL associated with CD revealed by acute intestinal obstruction. A North African woman of 38 years old with a history of infertility and chronic abdominal pain was admitted in emergency with acute intestinal obstruction. During the surgery, we found a tumor on the small intestine with mesenteric lymphadenopathy. Histology and immunohistochemistry of the specimen objectified a digestive T lymphoma CD3+ and immunological assessment of celiac disease was positive. The diagnosis of EATL was thus retained. Chemotherapy (CHOEP protocol) was established as well as gluten-free diet with a complete response to treatment. The EATL is a rare complication of CD that can be revealed by intestinal obstruction. The prognosis can be improved by early treatment involving surgery and chemotherapy. Its prevention requires early diagnosis of celiac and gluten-free diets.

## Introduction

Le lymphome T intestinal associé à une entéropathie ou Enteropathy-associated T-cell lymphoma (EATL) pour les anglo-saxons, est une complication rare de la maladie cœliaque (MC) [1–5]. Il s'agit d'un lymphome de mauvais pronostic du fait des complications au moment du diagnostic, de sa réponse aléatoire au traitement et du mauvais état nutritionnel des patients [2, 6]. Il est révélé parfois par un tableau d'abdomen aigu chirurgical [1, 2]. Nous rapportons l'observation d'un lymphome T associée à une MC, révélé par une occlusion intestinale aigue.

## Patient et observation

Patiente MS, Maghrébine, âgée de 38 ans, avec antécédent de stérilité primaire. L'anamnèse retrouvait des troubles du transit intestinal à type de constipation associée à des épigastralgies évoluant depuis 6 ans. L'examen physique ne notait rien de particulier. Une fibroscopie œso-gastro duodénale (FOGD) était indiquée et révélait une gastrite antrale chronique non atrophique et non active, sans métaplasie intestinale, ni *Hélicobacter pylori*. La patiente était mise sous inhibiteur de la pompe à proton (IPP) et antispasmodiques, sans amélioration clinique. Huit mois après la FOGD, la patiente était admise aux urgences chirurgicales pour occlusion intestinale aigue. L'intervention chirurgicale retrouvait une tumeur du grêle qui était reséquée et des adénopathies mésentériques qui étaient prélevées. Les suites opératoires étaient simples.

**Figure 1:**
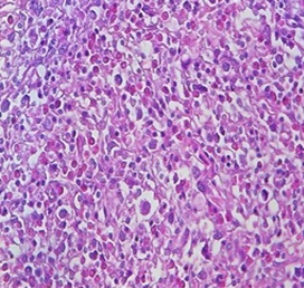
Lymphocyte T NOS: prolifération lymphomateuse (lymphocytes de grande taille)

**Figure 2:**
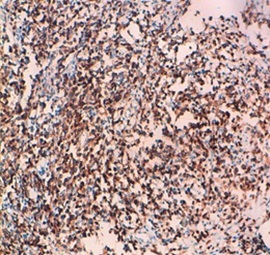
Lymphome T NOS forte expression du CD3 par les cellules tumorales

**Figure 3:**
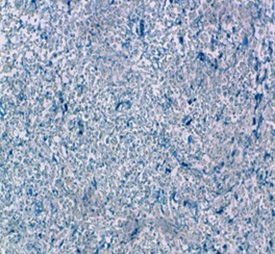
Lymphome T NOS absence d'expression du CD30 par les cellules tumorales

**Figure 4:**
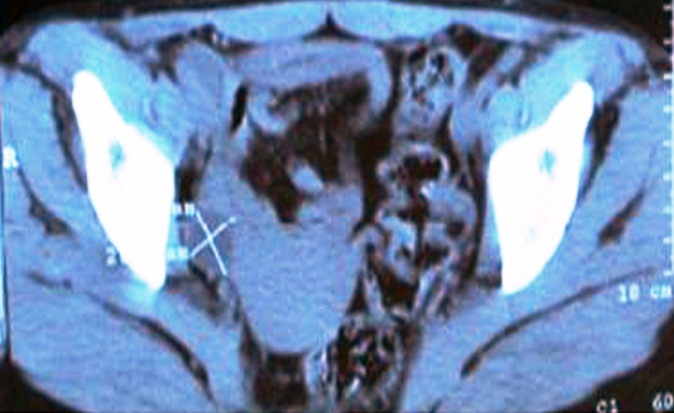
TDM abdominale montrant les adénopathies mésentériques

L'histologie et l'immuno-histochimie des pièces anatomo-pathologiques décelaient un lymphome T digestif (Figure 1) avec un profil phénotypique CD3+ (Figure 2), n'exprimant pas le CD20, le CD30 (Figure 3) et l'antigène épithélial membranaire (EMA). Une seconde FOGD avec biopsie duodénale objectivait une atrophie villositaire totale. La tomodensitométrie cervico-thoraco-abdomino-pelvienne (TDM CTAP) montrait la présence de plusieurs adénopathies (ADP) grossièrement arrondies cœlio-mésentériques, latéro-aortiques et au niveau de la bifurcation dont la plus grosse mesurait 2cm (Figure 4). Le bilan immunologique à la recherche de la maladie cœliaque était positif avec des anticorps Anti-endomysium IgA positif > 1/10 et anticorps anti-transglutaminase positif à 89,9UI /ml. La vitesse de sédimentation (VS) était à 54 mm à la 1ère heure. La C réactive protéine (CRP) était à 6,5mg/l. La numération formule sanguine (NFS), la lactate déshydrogénase (LDH), l'ionogramme sanguin et la fonction rénale étaient normaux. Les sérologies du virus de l'immuno-déficience humaine (VIH), des hépatites B et C étaient négatives.

La patiente était mise sous chimiothérapie avec le protocole CHOEP (cyclophosphamide, doxorubicine, vincristine, etoposide, prednisone) associé au régime sans gluten (RSG). La réponse était complète au 4ème cycle de CHOEP, objectivée par la disparition complète des troubles digestifs et des adénopathies à la TDM CTAP. La tolérance clinique et biologique au traitement était bonne. La patiente a ainsi pu bénéficier de 2 cures supplémentaires de CHOEP. Après 6 mois de suivi, la patiente ne présentait aucun signe de récidive avec reprise des activités.

## Discussion

La maladie cœliaque (MC) est une maladie auto-immune induite par le gluten, touchant entre 0,6 à 2% d'individus génétiquement prédisposés [4, 7–9]. La MC touche essentiellement les sujets de type caucasien et d'Afrique du Nord; elle est exceptionnelle chez les noirs et les asiatiques [10]. Les formes frustes, paucisymptomatiques, voire latentes sont nombreuses [11]. Notre patiente est une maghrébine chez qui la maladie cœliaque était méconnue, malgré une symptomatologie clinique évocatrice. Les arguments qui ont conduit à retenir le diagnostic de MC étaient: les antécédent de stérilité, les constipations et douleurs abdominales chroniques, la positivité des anticorps anti endomysium et anti transglutaminases, une atrophie villositaire duodénale totale et la disparition des signes cliniques sous régime sans gluten (RSG) [10, 12].

Le risque de transformation lymphomateuse T d'une MC, mieux connue sous l'acronyme anglais EATL (enteropathy associated T-cell lymphoma) est rare et survient dans environ 0,04% [2, 8]. Le mécanisme de survenue des EATL reste inconnu. L'augmentation de la perméabilité intestinale aux carcinogènes environnementaux, une inflammation chronique et les carences nutritionnelles induites par la MC seraient impliquées [9]. Le risque est d'autant plus élevé que le RSG est non ou mal suivi ou que le diagnostic de MC est tardif [12]. L’âge moyen au moment du diagnostic de l'EATL est de 60 ans avec des extrêmes de 33 et 81 ans et une prédominance masculine [1, 12–14].

Le délai moyen de survenue de l'EATL, lorsque la MC est connue au préalable, varie entre 5 à 10 ans [9, 13]. La découverte du lymphome se fait de façon concomitante avec la maladie cœliaque dans 20 à 70% des cas [13] Les symptômes les plus fréquents sont les douleurs abdominales, la diarrhée, l'anorexie, voire plus rarement un syndrome tumoral; mais dans plus de 40%, le mode de révélation de l'EATL est une urgence chirurgicale (perforation ou occlusion intestinale) [1, 2, 15]. L'occlusion intestinale a permis dans notre cas de découvrir le lymphome T associé à la MC. Dans 90% des cas, l'atteinte lymphomateuse T siège au niveau du grêle mais elle peut être multifocale [1] comme retrouvé chez notre patiente où l'atteinte du grêle était associée à une extension ganglionnaire. L'EATL est un lymphome T qui correspond à une prolifération monoclonale de lymphocytes intra-épithéliaux atypiques à grande cellules de « haut grade » [4]. L'OMS a individualisé deux types d'EATL [1]: le type I constituant 80% des EATL, associé à la maladie cœliaque dans 80 à 90% des cas et exprimant le CD3, mais négatif au CD56 et le type II représentant moins de 20% des cas exprimant le CD56 et le CD8. L'histologie de la pièce opératoire de notre patiente concluant à un lymphome T digestif exprimant le CD3 et associé à la maladie cœliaque; ce qui avait permis de retenir le diagnostic d'EATL type I.

La prise en charge de l'EATL n'est pas encore bien codifiée [2]. Le principal problème auquel le thérapeute est confronté est le mauvais état général des patients lié aux carences nutritionnelles prolongées. La nature très agressive et souvent chimio-résistante des EATL grève le pronostic [2]. Plusieurs protocoles de chimiothérapie ont été rapporté (CHOP, MOPP, BACOP, CHOEP, MACOPB, AC VB, CEOP, CHO IVE/MTX-ASCT), dont le plus préconisé est le CHOP [1, 13, 14]. Selon certains auteurs [14], la chirurgie s'impose uniquement en urgence pour traiter une complication (45 à 72% des interventions chirurgicales selon les séries) et n'est jamais thérapeutique seule. Van de Water [8], préconise de réaliser de préférence une chirurgie première aussi précocement que possible, puis de compléter par une chimiothérapie. Nijeboer [2] en 2015, a proposé de démarrer une chimiothérapie à base d'anthracycline, 2 à 5 semaines après l'intervention chirurgicale. Ce traitement doit être intensifié par de fortes doses de chimiothérapie, puis consolidée par le protocole BEAM (BCNU-carmustine, Etoposide, Aracytine, Melphalan) et une greffe autologue de cellules souches pour de meilleurs résultats. Une nouvelle molécule, le brentuximab vedotin (anti CD30), pourrait être prometteuse, en association avec la chimiothérapie conventionnelle comme traitement initial de l'EATL [2].

En dépit de toutes ces thérapies, l'EATL reste un lymphome de mauvais pronostic. Ce dernier est lié aux résistances au traitement, aux complications à type de perforation, d'hémorragie, de sepsis au moment du diagnostic ou au cours du traitement et aux rechutes locales, avec une survie cumulée à 5 ans entre 8 et 60%, en fonction de la capacité des patients à recevoir les doses additives de chimiothérapies [1, 2, 14]. Chez notre patiente, après exérèse chirurgicale, le protocole CHOEP était administré avec une réponse complète (RC) dès la 4^ème^ cure, maintenue à la fin de la 6ème cure. Cette réponse favorable pourrait s'expliquer par la prise en charge à un stade précoce (stade II2E Ann-Arbor modifié par Musshoff, la LDH et la CRP normales). En effet plus la LDH est élevée plus l'EATL est de mauvais pronostic du fait qu'elle est le reflet d'une masse tumorale importante (taille > 5cm), source de complications mécaniques, septiques et métaboliques; de même une CRP élevée s'explique par des lésions tissulaires extensives, d'ulcérations ou de perforations [1]. Il a été démontré qu'une RC n'est obtenue que chez 35 à 40% des patients traités par chimiothérapie et que l'obtention d'une RC est plus fréquente chez les malades en stade I/II, avec 50 à 80% de RC, contre 0 à 11% pour les stades III/IV [14].

Le régime sans gluten a été instauré en traitement complémentaire de la chimiothérapie et de la chirurgie avec disparition complète des troubles digestifs après 4 mois d'observation. Ce régime devra être maintenu définitivement afin de prévenir les rechutes et la survenue d'autres types de cancers associés à la maladie cœliaque [14].

## Conclusion

L'EATL est une complication rare et grave de la maladie cœliaque; elle peut être révélée par une occlusion intestinale aigue. Son traitement, encore non standardisé, associe la chirurgie précoce à une chimiothérapie intensive. Le pronostic de l'EATL reste sombre. Sa prévention passe par le diagnostic précoce de la MC et sa prise en charge conséquente par le régime sans gluten.

## References

[1] Delabie J, Holte H, Vose JM, Ullrich F, Jaffe ES, Savage KJ (2011). Enteropathy-associated T-cell lymphoma: clinical and histological findings from the international peripheral T-cell lymphoma project. Blood..

[2] Nijeboer P, Malamut G, Mulder CJ, Cerf-Bensussan N, Sibon D, Bouma G (2015). Enteropathy-associated T-cell lymphoma: improving treatment strategies. Dig Dis Basel Switz..

[3] Verbeek WHM, Van De Water JMW, Al-Toma A, Oudejans JJ, Mulder CJJ, Coupé VMH (2008). Incidence of enteropathy--associated T-cell lymphoma: a nation-wide study of a population-based registry in The Netherlands. Scand J Gastroenterol..

[4] Verkarre V, Brousse N (2013). Le diagnostic histologique de la maladie cœliaque. Pathol Biol..

[5] Nijeboer P, de Baaij LR, Visser O, Witte BI, Cillessen SAGM, Mulder CJ (2015). Treatment response in enteropathy associated T-cell lymphoma; survival in a large multicenter cohort. Am J Hematol..

[6] Biagi F, Gobbi P, Marchese A, Borsotti E, Zingone F, Ciacci C (2014). Low incidence but poor prognosis of complicated coeliac disease: a retrospective multicentre study. Dig Liver Dis Off J Ital Soc Gastroenterol Ital Assoc Study Liver..

[7] Lamireau T, Clouzeau H (2013). Épidemiologie de la maladie cœliaque. Pathol Biol..

[8] Van de Water JM, Nijeboer P, de Baaij LR, Zegers J, Bouma G, Visser OJ (2015). Surgery in (pre)malignant celiac disease. World J Gastroenterol..

[9] Catassi C, Bearzi I, Holmes GKT (2005). Association of celiac disease and intestinal lymphomas and other cancers. Gastroenterology..

[10] Malamut G, Cellier C (2010). Maladie cœliaque. Rev Médecine Interne..

[11] Lamireau T, Clouzeau H (2008). Comment confirmer le diagnostic de maladie cœliaque?. Arch Pédiatrie..

[12] Cosnes J, Nion-Larmurier I (2013). Les complications de la maladie cœliaque. Pathol Biol..

[13] Yasuoka H, Masuo T, Hashimoto K, Sato K, Okada S, Kusano M (2007). Enteropathy-type T-cell lymphoma that was pathologically diagnosed as celiac disease. Intern Med Tokyo Jpn..

[14] Chandesris M-O, Malamut G, Verkarre V, Meresse B, Macintyre E, Delarue R (2010). Enteropathy-associated T-cell lymphoma: a review on clinical presentation, diagnosis, therapeutic strategies and perspectives. Gastroenterol Clin Biol..

[15] Sato K, Uchiyama M (2011). Early radiological findings on CT in a patient with enteropathy-associated T cell lymphoma. BMJ Case Reports.

